# Unraveling the Pathogenesis of Asthma and Chronic Obstructive Pulmonary Disease Overlap: Focusing on Epigenetic Mechanisms

**DOI:** 10.3390/cells11111728

**Published:** 2022-05-24

**Authors:** Yung-Che Chen, Yu-Ping Chang, Kuo-Tung Huang, Po-Yuan Hsu, Chang-Chun Hsiao, Meng-Chih Lin

**Affiliations:** 1 Division of Pulmonary and Critical Care Medicine, Department of Medicine, Kaohsiung Chang Gung Memorial Hospital and Chang Gung University College of Medicine, Kaohsiung 83301, Taiwan; b9002087@cgmh.org.tw (Y.-P.C.); jelly@adm.cgmh.org.tw (K.-T.H.); wanpasum@cgmh.org.tw (P.-Y.H.); 2 Department of Medicine, College of Medicine, Chang Gung University, Taoyuan 33302, Taiwan; 3 Graduate Institute of Clinical Medical Sciences, College of Medicine, Chang Gung University, Taoyuan 33302, Taiwan

**Keywords:** asthma-chronic obstructive pulmonary disease overlap, epigenetics, DNA methylation, histone acetylation, microRNA

## Abstract

Asthma and COPD overlap (ACO) is characterized by patients presenting with persistent airflow limitation and features of both asthma and COPD. It is associated with a higher frequency and severity of exacerbations, a faster lung function decline, and a higher healthcare cost. Systemic inflammation in COPD and asthma is driven by type 1 T helper (Th1) and Th2 immune responses, respectively, both of which may contribute to airway remodeling in ACO. ACO-related biomarkers can be classified into four categories: neutrophil-mediated inflammation, Th2 cell responses, arachidonic acid-eicosanoids pathway, and metabolites. Gene–environment interactions are key contributors to the complexity of ACO and are regulated by epigenetic mechanisms, including DNA methylation, histone modifications, and non-coding RNAs. Thus, this review focuses on the link between epigenetics and ACO, and outlines the following: (I) inheriting epigenotypes without change with environmental stimuli, or epigenetic changes in response to long-term exposure to inhaled particles plus intermittent exposure to specific allergens; (II) epigenetic markers distinguishing ACO from COPD and asthma; (III) potential epigenetic drugs that can reverse oxidative stress, glucocorticoid insensitivity, and cell injury. Improved understanding of the epigenetic regulations holds great value to give deeper insight into the mechanisms, and clarify their implications for biomedical research in ACO.

## 1. Introduction

### 1.1. Asthma and Chronic Obstructive Pulmonary Disease Overlap Is Associated with Higher Frequency and Severity of Exacerbations and Higher Health Care Cost

Asthma and chronic obstructive pulmonary disease (COPD) are the two most common obstructive airway diseases. Asthma and COPD overlap (ACO) is characterized by patients presenting with persistent airflow limitation and features of both asthma and COPD. A recent meta-analysis demonstrates a high symptom burden, including dyspnea and wheezing, a lower lung function, and a higher frequency and severity of exacerbations associated with ACO compared with simple asthma and COPD [1]. Patients with ACO have increased disease severity, live a poorer quality of life, and incur higher healthcare costs when compared with patients with asthma or COPD alone [2,3]. The frequency of ACO-related hospitalization increases, the medical utilization rate increases, and the survival time is shortened compared with simple asthma and COPD [4,5]. Patients with ACO based on late onset asthma had the most rapid decline in lung function with a forced expiration volume at 1 s (FEV1) decline of 49.6 mL/year, compared with 39.5 mL/year in COPD alone, 34.5 mL/year in asthma alone, and 27.3 mL/year in ACO patients based on early onset asthma [6,7,8,9]. Patients with ACO had higher respiratory resistance and reactance during tidal breathing, but a smaller gap between the inspiratory and expiratory phases, compared with simple COPD [10]. ACO also had increased bronchial wall thickening, increased airway remodeling, and increased airway hyper-responsiveness compared with asthma and COPD patients [11,12]. ACO likely encompasses a wide spectrum of phenotypes, e.g., COPD with eosinophilia and partially reversible airflow limitation, severe asthma with neutrophilia and fixed airflow limitation, or elderly non-smokers with long-standing asthma and irreversible airflow limitation [3,4,13]. Although there is no universally accepted definition of ACO at present, it is recommended that ACO be defined based on the presence of persistent airflow limitation (post-bronchodilator FEV1/forced vital capacity (FVC) ratio < 70%) in symptomatic individuals 40 years of age and older, a well-documented history of asthma, probably before 40 years of age, and a significant exposure history to cigarette or biomass smoke, accompanied by one of the hyper-responsive features, including atopy, allergic rhinitis, eosinophilia, and positive bronchodilator response [14]. The global burdens of asthma and chronic obstructive pulmonary disease (COPD) are increasing. Each of which was estimated to affect approximately 339 million and 251 million people worldwide in 2016, while ACO may represent around 29.6% of COPD patients and around 26.5% of asthma patients [13].

### 1.2. Systemic Inflammation in COPD and Asthma Is Driven by Th1 and Th2 Immune Responses, Respectively, while Both Immune Responses May Contribute to Airway Remodeling in ACO

Asthma is usually characterized by airway hyper-responsiveness, leading to reversible airflow obstruction based on type 2 inflammation with eosinophilia. In contrast, COPD shows progressive and irreversible airflow obstruction, typically caused by smoking or biomass, and is associated with neutrophilic inflammation involving CD4/CD8 T lymphocytes and macrophages. Systemic inflammation is a feature of both COPD and asthma, and contributes to airway remodeling, airflow limitation, and subsequent symptoms. Inflammation in COPD is typically driven by T-helper (Th) 1 immune responses, which enhance cell-mediated immunity and phagocyte-dependent inflammation through the production of interleukin (IL)-2, IL6, IL-8, IL-9, IL-17A, interferon-γ, and tumor necrosis factor (TNF)-α [15,16]. Pro-inflammatory cells, such as dendritic cells, neutrophils, and macrophages, are recruited to small airways and functionally altered by oxidative stress, intracellular activation of the transcription factor nuclear factor-κB (NF-κB), and defective bacterial phagocytosis, resulting in airflow limitation [16,17]. Key players of inflammatory response in asthma are Th2 and type 2 innate lymphoid cells, which produce IL-4, IL-5, IL-6, IL-9, IL-13, and IL-17E, resulting in overt immunoglobulin E (IgE) production, eosinophil accumulation, and inhibition of phagocyte-independent inflammation [15,17,18]. The two different inflammatory mechanisms involved in asthma and COPD may overlap in ACO patients. Studies have shown that ACO patients have higher fractions of exhaled nitric oxide (FeNO), higher blood eosinophil counts and percentages, and higher Th2 inflammation markers than COPD patients, as well as increased total and specific IgE levels [2,13,19,20]. However, similarities and differences between specific gene expression profiles in ACO, asthma, and COPD have not yet been studied, but could add to our understanding of the biology underlying the clinical and pathologic overlap between asthma and COPD.

## 2. Potential Biomarkers for ACO

Table 1 lists important molecules and their roles in the pathology and clinical phenotypes of ACO. 

### 2.1. Neutrophilic Inflammation-Related

TNF-α regulates inflammatory cell functions such as cell proliferation, survival, differentiation, and apoptosis, and its genetic variant TNF-α-308 GA contributes to the susceptibility to COPD. Blood TNF-α concentrations of ACO patients were higher than that of asthmatics, but lower than that of pure COPD patients [21,22]. On the other hand, TNF-α was up-regulated in ACO mice versus either COPD or asthma mice [23]. As an anti-inflammatory cytokine, IL-10 has the capacity to suppress excessive inflammation by increasing M2 polarization and decreasing neutrophil infiltration. IL10 was down-regulated in ACO patients versus COPD [22]. Likewise, cathelicidin antimicrobial peptide (CAMP; LL37) was down-regulated in ACO patients versus COPD, while it not only directly recruits immunocompetent cells but also inhibits pro-inflammatory cytokine synthesis [24].

Neutrophil gelatinase-associated lipocalin (NGAL) is abundantly secreted by neutrophils and other immune cells during bacterial infections to hamper bacterial growth through restriction of iron availability and contributes to activation of iron-responsive genes like ferritin and transferrin receptors. NGAL protein levels have been shown to be elevated in sputum or serum samples from ACO patients versus COPD or asthma [25]. Given that NGAL has been demonstrated to be a good marker for early diagnosis of acute kidney injury and the prognosis of colon and breast cancers, it is less practicable to use this single marker to discriminate ACO from COPD or asthma [26]. 

Spleen associated tyrosine kinase (SYK), a 72kD non-receptor protein tyrosine kinase, can trigger cell degranulation and histamine release in human basophils through FcεRI-mediated signaling pathways and is required for neutrophils to form neutrophil extracellular traps. Phosphorylated SYK binds to the ITAM, triggering its downstream inflammatory signal cascades, including NF-κB and NLRP3, while the specific SYK inhibitor, EAPP-2, significantly down-regulates the expression of NF-κB, phosphorylated-NF-κB, and NLRP3 in ACO mice and in LPS-induced RAW264.7 macrophages in vitro [27].

### 2.2. Th2 Response-Related

In an allergic inflammatory microenvironment, pro-inflammatory cytokines and oxidative stress might up regulate the production of nitric oxide (NO) synthetase-2-derived NO, which produces strong oxidizing reactive nitrogen species, such as peroxynitrite, leading to cell damage in the airways [28]. IgE is synthesized by lymphocytes B upon IL-4-induced Ig class switching, and causes allergic inflammation process through interacting with dendritic cells, mast cells, eosinophils, airway epithelial cells and airway smooth muscle cells [20]. Periostin is produced by airway epithelial cells in response to type2 cytokines IL4 and IL13 stimuli, and augments eosinophilic inflammation through the αMβ2 integrin and the generation of a superoxide anion [29]. IL5 promotes recruitment and survival of eosinophils in airways as well as maturation of granules and contributes to bronchoconstriction, allergic response, fibrosis, epithelial injury, and oxidative stress. Fractional exhaled NO (FENO), IgE, serum periostin, and serum IL5, which are typical biomarkers of Th2 or eosinophilic responses, have all been reported to be able to distinguish ACO from COPD [29,30,31,32]. There are some differences in Th2 signatures between the two main types of ACO. Asthmatic smokers with chronic airflow obstruction (post-bronchodilator FEV1/FVC ≤ 0.7) had higher blood eosinophil counts than but similar FENO to simple COPD patients, while COPD patients with eosinophilia (≥200 eosinophils·μL^−1^) had higher FENO and blood eosinophil counts than both asthmatic smoker with chronic airflow obstruction and simple COPD patients [33].TBX21 and GATA3 have conventionally been regarded as a transcription factor that drives the differentiation of Th1 and Th2 cells, respectively, and increased TBX21/GATA3 gene expression ratios were found in ACO patients versus COPD or asthma [34,35]. IL-6 is a pleotropic cytokine produced in response to tissue damage, and might favor Th2 and Th17 polarization by stimulating STAT3 [36]. IL-6 was up-regulated in both ACO patients and ACO mice versus COPD or asthma [22,23]. IL-17E also induces allergic inflammation in favor of Th2 immune response. IL-17E was up-regulated in ACO patients versus COPD [22,37].

Eosinophil-derived neurotoxin (EDN) is one of the four specific granules of the human eosinophilic leukocyte and is released when these cells are activated by Th2 cytokines. EDN has been determined to be a specific biomarker in eosinophil-associated pathophysiologies, including asthma exacerbations, cow’s milk allergy, and eosinophilic esophagitis [38]. Recently, serum EDN was found to be higher in ACO patients than that in asthma or COPD patients and had the highest specificity (82.4%) when combined with high serum YKL-40 levels based on cutoff values derived by receiver operating characteristics analysis (EDN: 23.0 ng/mL; YKL-40: 61.3 ng/mL) [39].

### 2.3. Arachidonic Acid-Eicosanoid Pathway-Related

Arachidonic acid is an essential unsaturated fatty acid and is metabolized to eicosanoids and bioactive lipid mediators, which augment type 2 inflammatory responses in the airway, including potent bronchoconstriction, recruiting, and activating T cells, eosinophils, and antigen presenting cells, promoting the secretion of mucus, promoting the proliferation of human airway epithelial cells and smooth muscle cells, and increasing collagen deposition. Several eicosanoids metabolized through lipoxygenase, including TETE, HPETE, and HPODE, in serum have been shown to be able to distinguish ACO from COPD [40]. Prostaglandin D2 synthase (PGD2) has been reported to be higher in asthma and ACO patients than in COPD patients [32].

### 2.4. Metabolites-Related

Metabolites are produced during normal endogenous metabolism within biofluids, cells, tissues, or organisms, and the ever-expanding metabolomics approach has provided new insight into mechanistic changes associated with various diseases. Five serum metabolites, including L-serine, L-threonine, ethanolamine, D-mannose, and succinic acid, were found to be significantly decreased in ACO as compared with asthma and COPD patients, providing new insights into the altered pathways which could be contributing to the higher mortality and morbidity in ACO [22]. Another 12 metabolites, including lipid, isoleucine, N-acetylglycoproteins, valine, glutamate, citric acid, glucose, L-leucine, lysine, asparagine, phenylalanine, and histidine, were dysregulated in ACO patients when compared with both asthma and COPD [41]. Isopropanol and acetone were increased in exhaled breath condensate from ACO patients versus COPD or asthma, while valine was decreased [42]. Many study groups have reported altered expressions of various metabolites in respiratory diseases, so it is an emerging research area with a large potential for developing novel biomarkers. However, the underlying mechanisms by which these metabolites correlate with neutrophil-mediated inflammation or Th2 responses in ACO require further investigation. 

## 3. Genetic Variants Associated with ACO

Elucidating the genetic determinants of ACO may illuminate disease mechanisms and aid in the early identification of high-risk patients. A polymorphism (rs4795405) in *ORMDL3* is associated not only with the development of asthma but also with COPD, suggesting a genetic overlap between these two diseases [46]. There is a significant overall genetic correlation of COPD with asthma in subjects with European ancestry and one genome-wide significant association in *KIAA1958* (rs59289606) has been identified by a genome-wide association study (GWAS), suggesting that childhood asthmatics are at increased risk for COPD [47]. In another GWAS, an intergenic single nucleotide polymorphism on chromosome 7 (rs111720447, C/A), which is located near glucocorticoid receptor binding sites, has been shown to be strongly associated with lung function decline in the inhaled corticosteroid (ICS) treatment group of COPD patients [48]. The COPDGene study showed that the single nucleotide polymorphism that was most strongly related to ACO in the combined ethnic groups was located on chromosome 14 near or within the gene *G-protein coupled receptor 65* (*GPR65*; rs6574978). Genetic knock-out of the *GPR65* gene has been shown to reduce levels of eosinophils in murine models of asthma [49,50]. However, most studies to date have been underpowered to detect genetic variants at a genome-wide significance level, and replication of genetic “hits” has been lacking.

## 4. Epigenetic Markers Associated with ACO

Table 2 lists selected epigenetic markers and their potential mechanisms in the pathology of ACO.

### 4.1. Aberrant DNA Methylation

Gene–environment interactions are key contributors to the complexity of ACO and are regulated by epigenetic mechanisms, including DNA methylation, histone modifications, and non-coding RNAs. Gene promoter DNA methylation, mediated by the DNA methyltransferase enzymes, allows permanent or temporary silencing of genes, and this commonly occurs across cytosine–guanine dinucleotide (CpG) methylation sites in the mammalian genome. DNA hyper methylation in promoter regions can lead to decreased transcription of the downstream genes, resulting in the inactivation of some key immune-related genes, while DNA methylation in the gene body is generally positively correlated with gene expression. In a pilot study, older smokers with asthma were associated with hypermethylation of selected genes, such as the *protocadherin-20* gene, in sputum DNA [51]. *Protocadherin-20* functions as a tumor-suppressor gene through antagonizing the Wnt/beta-catenin signaling pathway, which is involved in the lung injury-repair processes of chronic lung diseases [52,53]. 

In our recent epigenome-wide associated study analysis, we found that ACO patients had hypermethylated *phosphodiesterase 9A* (*PDE9A*; +30,088 CpG site)/*zinc finger and SCAN domain containing 31* (*ZNF323*; *ZSCAN31*;·−296 CpG site), and hypomethylated *septin 8* (*SEPT8*; −47 CpG site) genes as compared with either pure COPD patients or healthy non-smokers. Among these three DNA methylation patterns that showed to be the most significantly associated with ACO, only *ZNF323* gene methylation (−264 CpG site) was altered in response to cigarette smoke extract CSE plus ovalbumin OVA treatment in human monocytic THP-1 cells, while the other two gene methylations may be inheriting epigenotypes rather than changes after cigarette smoke exposures. PDE9A inhibitor, BAY-73-6691, can reduce neutrophil adhesion to the vessel wall through decreasing surface expressions of the L-selectin and CD11b adhesion molecules via elevating leukocyte cGMP levels, which in turn activates protein kinases that perform several regulatory functions, including smooth muscle relaxation, neuronal transmission, and inhibition of platelet aggregation [54]. SEPT8, a cytoskeleton protein, is involved in renal cellular organization and structure in response to hypoxic stress and involved in dysfunction and loss of synapses in Alzheimer’s disease through modulating beta-amyloidogenic processing of amyloid precursor protein by decreasing BACE1 levels [55,56]. Given that PDE9A and SEPT8 function in various biological processes of inflammation and cell cytokinesis/migration, respectively, they may be novel targets for ACO. ZNF323 contributes to the production of various catecholamines via augmenting tyrosine hydroxylase expression [57]. In line with our finding that hypermethylation of the *ZNF323* promoter region (−296 CpG site) was associated with a rapid decline in FEV1, several single nucleotide polymorphisms of the *ZNF323* gene have been shown to be associated with lung function in asthmatic patients, suggesting that this DNA methylation change may be another reversible therapeutic target for ACO. Additionally, we found that hypermethylated *mitochondrial inner membrane protein like* (*MPV17L*; +194 CpG site) gene was associated with rapid lung function decline in all the COPD patients, while in vitro demethylation agent, 5-aza-2′-deoxycytidine, treatment in THP-1 cells reversed CSE and OVA co-exposure-induced promoter hypermethylation-mediated *MPV17L* under-expression, as well as ameliorated cell apoptosis and oxidative stress. *MPV17L* can protect against mitochondrial oxidative stress and apoptosis by activation of Omi/HtrA2 protease, and its methylation has been shown to be associated with the prognosis of lung adenocarcinoma. Our results suggest that perturbation of *MPV17L* signaling through epigenetic programming may be a novel strategy to inhibit oxidative stress-induced cell injury in ACO [58].

### 4.2. Histone Modification Patterns and Histone Modifying Enzymes

Histones are the core proteins that wrap the DNA of a eukaryotic cell into nucleosomes. Each histone protein H3/H4 can be regulated by post-translational modifications in various ways, including acetylation, methylation, ubiquitination, and phosphorylation. Histone acetylation is mediated by acetyltransferases, which activate gene transcription, and histone deacetylases (HDACs), which suppress the transcription. Since over a thousand proteins are reversibly acetylated, and acetylation critically influences aberrant intracellular signaling pathways in asthma and COPD, HDACs that regulate posttranslational protein acetylation are promising targets for ACO. Decreased expressed HDAC2 have been noted both in severe COPD and uncontrolled asthma, and abolishes the effect of glucocorticoids by keeping glucocorticoid receptor from deacetylation and unable to form a protein–protein complex that represses the NF-κB pathway [59,60,61]. Additionally, reduced HDAC2 protein expression has been demonstrated in ACO mice by enhancing the phosphorylation of the Akt in PI3K-delta/Akt signaling pathway, and can be improved by a macrolide intervention, roxithromycin [62].

### 4.3. MicroRNA Dys-Regulations

Among human genes, 70% comprise the actively transcribed genome, less than 3% are protein-coding genes, and most of them are non-coding RNAs. Unlike messenger RNA, non-coding RNA functions either in the nucleus by binding to DNA to contribute to gene silencing, or in the cytoplasm by regulating mRNA to affect protein expression. MicroRNAs (miRNAs) are short non-coding RNA molecules of approximately 22 nucleotides that negatively regulate gene expression at the post-transcriptional level by degrading or binding to the 3’ untranslated region of target messenger RNAs. Several miRNAs have been shown to be up- or down-regulated in both asthma and COPD, such as miR125b, miR-155-5p, miR-21-5p, miR-218-5p, and miR-223 [63,64,65,66,67,68,69]. Although miR-223 is up-regulated in multiple samples from both asthma and COPD patients, it mainly acts as an anti-inflammatory miRNA by directly targeting multiple genes that are involved in the NF-kB pathway, such as PARP1, IKKa, TRAF6, CUL1, and TAB2. On the other hand, miR-223 also targets HDAC2, which is important for corticosteroid sensitivity. The subsequent reduction in HDAC2 activity in COPD or asthma could lead to lower corticosteroid sensitivity and less inhibition of pro-inflammatory cytokines and chemokines in response to corticosteroid treatment [70,71]. Previous studies showed that miR-185a-5p and miR-320a were both down-regulated in ACO patients versus asthma, while miR-320c and miR-1246 were both up-regulated [63,72]. However, these miRNAs could not distinguish ACO from COPD, and thus are not good biomarkers for ACO. In a recent study, miR-15b-5p, miR-19b-3p, miR-148a-3p and miR-26b-5p were all down-regulated in plasma from ACO patients versus either COPD or asthma. Among them, miR-15b-5p showed the best performance for ACO diagnosis, and has predicted target genes associated with pathways implicated in both asthma and COPD, such as AKT3, E2F3, MAP2K1, MAPK8, PIK3R1, RAF1, and VEGFA [73]. miR-19b-3p can protect endothelial cells from sepsis-induced inflammation injury via inhibiting NF-κB signaling pathway through directly targeting KLF7 [74]. miR-148a-3p can repress IKBKB/NF-κB signaling, and inflammatory gene expression in necrotizing enterocolitis via targeting Tp53 [75]. miR-26b-5p can inhibit cerebral ischemia / reperfusion-induced apoptosis and inflammatory responses via targeting Smad1 [76]. In another study, miR-619-5p and miR-4486 were both down-regulated in serum from eosinophilic ACO patients versus either COPD or asthma, while both miRNAs participate in two common pathways, metabolism of xenobiotics by cytochrome P450 and ErbB signalings, suggesting that targeting the EGFR signaling pathway or NRF2 modulators could be used as a novel therapeutic approach for ACO [77]. In contrast, miR-125b-5p, which has been shown to be involved in neutrophil activation and NF-κB-mediated inflammation via targeting TNFAIP3, was up-regulated in plasma from ACO patients versus COPD [63,78]. 

## 5. Limitations and Perspectives of Epigenetic-Related Investigations in ACO

Because epigenetic changes are inheritable and reversible, they represent not only a biomarker with great potentialities but also an attractive target for pharmaceutical intervention in ACO. However, several limitations should be acknowledged. First, the ACO animal model has not yet been fully and thoroughly established, and the evaluation of therapeutic drugs for ACO, including agents targeting epigenetic marks, is still in its infancy. However, a mouse model that can better simulate the clinical–pathological characteristics of ACO has been established recently and suggest that the whole-genome gene expression changes of ACO reflect biology beyond Th2 inflammation and are mainly driven by HLA-DRA, SYK, CTLA4, VAV1, NRAS, and JAK3 signaling pathways [23]. Second, patients with ACO are excluded from the majority of asthma and COPD randomized control trials, which contributes to the lack of efficacy and safety data on effective therapies for ACO. This, in combination with a limited number of specific interventional studies in ACO cohorts, has resulted in a lack of knowledge of effective treatment strategies and diagnostic/predictive/prognosis biomarkers for ACO. A recent meta-analysis combining three cohort studies showed that ICS/long-acting β2-agonist treatment was associated with a lower risk of death or hospitalization in ACO patients with a relative risk of 0.82, while long-acting β2-agonist was associated with decreased risk of myocardial infarction with a relative risk of 0.8 [81]. The only factor associated with a decrease in ACO exacerbation after ICS use was a blood eosinophil count of >/=300 cells/muL [82]. For the first time, a recent large-scale, multicenter, randomized controlled trial has demonstrated that the addition of long-acting muscarinic antagonist to ICS+long-acting β2-agonist is beneficial for the treatment of ACO patients in terms of lung function improvement but not in exacerbation or symptom control [83]. Although limited clinical data exist on the efficacy of biological agents in ACO, the Australian Omalizumab Registry’s study suggests that omalizumab, an anti-IgE monoclonal antibody, can improve symptoms and health-related quality of life in individuals with ACO [84,85]. Furthermore, meta-analyses of the randomized controlled trials showed that anti-IL-5 monoclonal antibodies, mepolizumab and benralizumab, could reduce annual moderate/severe exacerbation rates in eosinophilic COPD patients [86,87]. Third, reduced HDAC2 activity leads to lower steroid sensitivity in either asthma or COPD, and can be reversed by theophylline, roxithromycin, or miR-223 knock-down. Theophylline improves steroid sensitivity in both COPD and asthma and thereby decreases oxidative stress by increasing the expression and activity of HDAC2 [88,89]. However, none of the effects of the three treatments has been evaluated in ACO patients. Frequent gastrointestinal and cardiovascular side effects of theophylline are another concern when it is applied to ACO treatment. Fourth, several miRNAs have been shown to be promising biomarkers for ACO, such as miR-15b-5p, miR-125b-5p, miR-4486, and miR-619-5p. However, corresponding gene expression changes of their predicted target genes have not yet been verified in ACO patients, and their biological functions have yet to be examined in vitro. Finally, although the de-methylation agent, 5-aza-2′-deoxycytidine, has been shown to reverse promoter hypermethylation-mediated *MPV17L* under-expression in response to cigarette smoke extract and ovalbumin co-exposure, and reverse cell apoptosis and over-production of reactive oxygen species, it is not a specific agent targeting *MPV17L*. Further investigation of gene-specific demethylation by using site-specific methylome editing is required for clinical application to ACO treatment.

## 6. Conclusions

**Figure 1:**
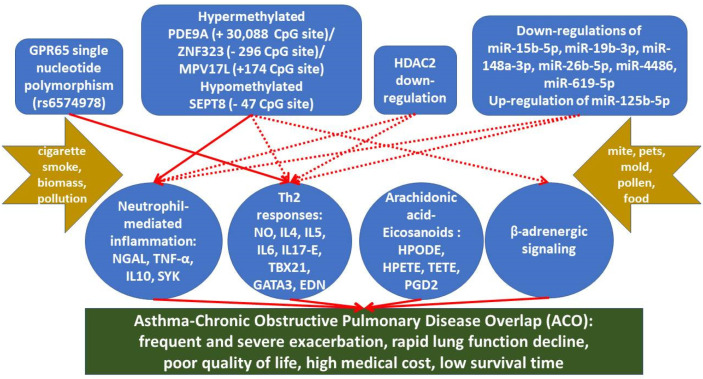
Proposed model of the roles of epigenetics-mediated regulations in the development of asthma and chronic obstructive pulmonary disease overlap (ACO) and its clinical phenotypes.

**Figure 2:**
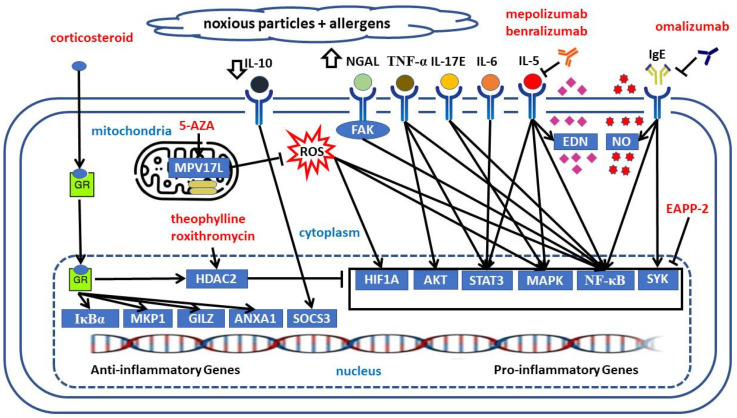
Potential medicines, including epigenetic drugs, and their actions on regulating pro-inflammatory or anti-inflammatory responses in asthma and chronic obstructive pulmonary disease overlap.

In this review, we have highlighted the growing importance of the epigenetic regulation of neutrophil-mediated inflammatory and Th2 responses to cigarette smoke and allergen exposures in ACO. Figure 1 shows a proposed model of the epigenetic regulations in ACO based on the results of the cohort studies, in vitro experiments, or animal studies. Figure 2 shows potential epigenetic drugs, important medicines that have been proved to be effective in both asthma and COPD or ACO, and their actions on regulating pro-inflammatory or anti-inflammatory responses in ACO. We need to understand this heterogeneity and mechanisms that underlie systemic responses to long-term exposures to cigarette smoke or biomass and intermittent environmental exposure to allergens in the same or reverse sequence, or concomitantly. In this regard, of particular interest is the potential for epigenetic mechanisms to regulate neutrophilic or eosinophilic inflammation, the imbalance between Th1 and Th2 response, oxidative stress, airway remodeling, and β2-adrenergic signaling that can affect bronchospasm directly. Epigenetic regulation of immune and respiratory systems is an important area for future research in ACO.

Continuing technological advances would enable a deeper understanding of the epigenetic mechanisms that control the recruitment, polarization, and activation of immune cells in response to cigarette smoke and allergen co-stimuli at single-cell resolution, and their application in combination with multi-omic strategies to ACO has the potential to identify cell- and tissue-specific epigenetic targets for disease treatments across organs. Integrating all genome and different types of epigenetic alteration data may help to reveal the regulatory role of epigenetics in the formation of ACO more comprehensively [90]. Clinically, the evaluation and modulation of epigenetics manifest as promising prospects in the diagnosis and treatment of ACO. Epigenetic drugs with new targets have gradually entered the horizon of researchers in recent years. Demethylation agent, 5-aza-2′-deoxycytidine, treatment could reverse CSE and OVA co-exposure-induced *MPV17L* under-expression, oxidative stress, and cell injury, while theophylline and roxithromycin could overcome HDAC 2 under-expression observed in both COPD and asthma. These findings provide initial evidence to support the potential effectiveness of the epigenetic agents for ameliorating lung function decline, exacerbation, and symptom burdens in ACO, while gene-editing by using clustered regularly interspaced short palindromic repeat (CRISPR) to deliver the epigenetic drug to a specific genomic locus will serve as a key technique in the future [91]. Overall, only limited studies have focused on the involvement of epigenetic regulations in ACO, and further investigations are still needed.

Continuous lines and arrows represent proposed cause and effect relationships based on the findings from the cohort studies, in vitro experiments, or animal models, while dotted lines and arrows represent hypothetical relationships not approved by specific investigations in ACO.

Arrows represent a positive action on the target, whereas bar-headed arrows represent an inhibition.

5-AZA = 5-aza-2′-deoxycytidine; AKT = AKT serine/threonine kinase 1; ANXA1 = annexin A1; EDN = eosinophil-derived neurotoxin; FAK = protein tyrosine kinase; GILZ = TSC22 domain family member 3; GR = glucocorticoid receptor; HDAC2 = histone deacetylase 2; HIF1A = hypoxia inducible factor 1 subunit alpha; IκBα = NFKB inhibitor alpha; IgE = immunoglobulin E; IL = interleukin; MAPK = mitogen-activated protein kinase; MKP1 = dual specificity phosphatase 1; MPV17L = MPV17 mitochondrial inner membrane protein like; NGAL = lipocalin 2; NO = nitric oxide; ROS= reactive oxygen species; SOCS3 = suppressor of cytokine signaling 3; STAT3 = signal transducer and activator of transcription 3; SYK = spleen associated tyrosine kinase; TNF-α = tumor necrosis factor.

## Figures and Tables

**Table 1:** Important molecules and their roles in the pathology and clinical phenotypes of asthma and chronic obstructive pulmonary disease overlap (ACO).

	Expression Levels in ACO	Investigation Model	Main Role	Reference
Neutrophil-mediated inflammation				
neutrophil gelatinase-associated lipocalin (NGAL)	Increased vs. COPD or asthma	Serum/sputum; ACO rats	Positively correlated with blood eosinophil counts, negatively correlated with FEV1 & FEV1/FVC	[25,39,43,44]
TNF-α	Increased vs. COPD or asthma	Serum; ACO mice		[21,22,23]
IL10	Decreased vs. COPD	serum		[22]
LL37	Decreased vs. COPD	sputum	Negatively correlated with FEV1, FEV1/FVC, & sputum neutrophil counts	[45]
spleen associated tyrosine kinase (SYK)	Increased	ACO mice	Required to form neutrophil extracellular traps; inducing IL-1β, IL-6, and TNF-α via activating NF-κB.	[23,27]
Th2 responses				
nitric oxide	Increased vs. COPD	Exhaled fraction	correlated with FEV1 %predicted	[31]
periostin	Increased vs. COPD	serum	Positively correlated with blood eosinophil counts and total IgE	[29]
IL4	Increased vs. COPD or asthma	ACO mice		[23]
IL5	Increased vs. COPD or asthma	serum	Negatively correlated with FEV1 & FEV1/FVC	[22]
IL6	Increased vs. COPD or asthma	Serum; ACO mice	Negatively correlated with FEV1 & FEV1/FVC	[22,23]
IL17-E	Increased vs. COPD	serum		[22]
TBX21/GATA3	Increased gene expression ratios in ACO vs. COPD or atopic asthma	Peripheral blood mononuclear cells		[35]
eosinophil-derived neurotoxin (EDN)	Increased vs. COPD or asthma	serum	Positively correlated with blood eosinophil counts, negatively with FEV1/FVC	[39]
Arachidonic acid-Eicosanoids pathways				
HPODE	Increased vs. COPD	serum	Negatively correlated with FEV1/FVC	[40]
HPETE	Increased vs. COPD	serum	Negatively correlated with FEV1/FVC	[40]
HETE	Increased vs. COPD	serum	Negatively correlated with FEV1/FVC	[40]
prostaglandin D2 (PGD2)	Increased vs. COPD	serum	Negatively correlated with FEV1/FVC% values	[32]

HPODE = hydroperoxy-octadecadienoic acid; HPEDE = hydroperoxy-eicosadienoic acid; HETE = hydroxy-eicosatetraenoic acid.

**Table 2:** Selected epigenetic markers and their potential mechanisms in the pathology of asthma and chronic obstructive pulmonary disease overlap (ACO).

Epigenetic Markers	Changes in ACO	Investigation Model	Potential Mechanisms	Reference
PDE9A	Hypermethylated gene body (+30088) in ACO vs. COPD or HS	PBMCs	Augmenting neutrophil adhesion by hydrolysis of cGMP	[54,58]
SEPT8	Hypomethylated gene promoter (−47) in ACO vs. COPD or HS	PBMCs	Augmenting cytokinesis and migration of immune cells	[58,79]
ZNF323	Hypermethylated gene promoter (−296) in ACO vs. COPD or HS	PBMCs	Inhibiting catecholamine synthesis by decreasing tyrosine hydroxylase activity	[57,58]
MPV17L	Hypermethylated gene promoter (+174) in ACO vs. healthy subjects	PBMCs; THP1 cell under co-exposure of cigarette smoke extract and ovalbumin	Augmenting mitochondrial oxidative stress and apoptosis	[58,80]
HDAC2	Decreased expression	ACO mice	Desensitizing glucocorticoid receptor	[59]
miR-15b-5p	Down-regulated in ACO vs. COPD or asthma	serum	Targeting AKT3, E2F3, MAP2K1, MAPK8, PIK3R1, RAF1, and VEGFA	[72]
miR-19b-3p	Down-regulated in ACO vs. COPD	serum	Inhibiting NF-κB signaling via targeting KLF7	[63,74]
miR-125b-5p	Up-regulated in ACO vs. COPD	serum	Promoting NF-κB-mediated inflammation via targeting TNFAIP3	[63]
miR-148a-3p	Down-regulated in ACO vs. COPD or asthma	serum	Inhibiting IKBKB/NF-κB signaling via targeting Tp53	[72,75]
miR-26b-5p	Down-regulated in ACO vs. COPD or asthma	serum	Inhibiting inflammation via targeting SMAD1	[72,76]
miR-4486	Down-regulated in ACO vs. COPD or asthma	serum	Targeting ERBB2	[77]
miR-619-5p	Down-regulated in ACO vs. COPD or asthma	serum	Targeting ERBB2	[77]

PBMC = peripheral blood mononuclear cell.

## Data Availability

Not applicable.

## Abbreviations

COPD	chronic obstructive pulmonary disease
ACO	asthma and COPD overlap
FEV1	forced expiratory volume at 1 s
FENO	fractional exhaled nitric oxide
FVC	forced vital capacity
Th	T helper
IL	interleukin
TNF	tumor necrosis factor
NF-κB	nuclear factor kappa B
Ig	immunoglobulin
NGAL	neutrophil gelatinase-associated lipocalin
SYK	spleen associated tyrosine kinase
NO	nitric oxide
EDN	eosinophil-derived neurotoxin
PGD2	prostaglandin D2 synthase
GPR65	G-protein coupled receptor 65
CpG	cytosine–guanine dinucleotide
PDE9A	phosphodiesterase 9A
SEPT8	septin 8
ZNF323/ZSCAN31	zinc finger and SCAN domain containing 31
MPV17L	MPV17 mitochondrial inner membrane protein like
HDAC	histone de-acetylase
miRNA	microRNA
